# Methodological considerations in a pilot study on the effects of a berry enriched smoothie on children’s performance in school

**DOI:** 10.1080/16546628.2017.1409063

**Published:** 2017-11-30

**Authors:** Ulla Rosander, Kimmo Rumpunen, Viktoria Olsson, Mikael Åström, Pia Rosander, Karin Wendin

**Affiliations:** ^a^ School of Education and Environment, Kristianstad University, Kristianstad, Sweden; ^b^ Department of Plant Breeding, Swedish University of Agricultural Sciences, Balsgård, Sweden; ^c^ Department of biostatistics, StatCons, Malmö, Sweden; ^d^ Department of Food Science, University of Copenhagen, Copenhagen, Denmark

**Keywords:** Berries, beverage, concentration, d2 test of attention, fruits, total error percentage, vegetables

## Abstract

Berries contain bioactive compounds that may affect children’s cognitive function positively, while hunger and thirst during lessons before lunch affect academic performance negatively. This pilot study addresses methodological challenges in studying if a berry smoothie, offered to schoolchildren as a mid-morning beverage, affects academic performance.

The objective was to investigate if a cross-over design can be used to study these effects in a school setting.

Therefore, in order to investigate assay sensitivity, 236 Swedish children aged 10–12 years were administered either a berry smoothie (active) or a fruit-based control beverage after their mid-morning break. Both beverages provided 5% of child daily energy intake. In total, 91% of participants completed the study. Academic performance was assessed using the d2 test of attention. Statistical analyses were performed using the Wilcoxon signed rank test in StatXact v 10.3.

The results showed that the children consumed less of the active berry smoothie than the control (154 g vs. 246 g). Both beverages increased attention span and concentration significantly (p = 0.000). However, as there was no significant difference (p = 0.938) in the magnitude of this effect between the active and control beverages, the assay sensitivity of the study design was not proven. The effect of the beverages on academic performance was attributed the supplementation of water and energy.

Despite careful design, the active smoothie was less accepted than the control. This could be explained by un-familiar sensory characteristics and peer influence, stressing the importance of sensory similarity and challenges to perform a study in school settings. The employed cross-over design did not reveal any effects of bioactive compound consumption on academic performance. In future studies, the experimental set up should be modified or replaced by e.g. the parallel study design, in order to provide conclusive results.

## Introduction

Children, along with the malnourished and the elderly, are particularly sensitive to the effects of diet on performance [1]. Brain function is sensitive to short-term variations in nutrient supply, with several studies examining the immediate (short-term) effects of breakfast on academic performance [2,3]. However, research investigating the long-term effects of breakfast in this regard is less common in the literature [4]. Nonetheless, evidence suggests that eating breakfast can improve learning in children in terms of behaviour, cognitive performance and school performance [3,5,6], especially in children at nutritional risk [7]. However, not all children eat breakfast and even if breakfast is consumed, the positive effects may not last, with a Swedish study reporting that 80% of schoolchildren were hungry an hour before lunch [8]. Although the consumption of a mid-morning snack may maintain or improve attention and academic performance throughout the morning, literature regarding the impact of snacks on cognition is very limited [9] and the meal effect may be different depending on whether a nightly rest or a working phase took place beforehand [10].

Providing children with a drink of water and thereby counteracting dehydration may be enough to affect performance in cognitive tasks [11]. Further, to maintain their higher metabolic rate, children require a continuous supply of energy derived from glucose [2]. However, water and energy are not the only dietary components that play a role; in addition to carbohydrates, fruits and vegetables contain vitamins, minerals and other bioactive substances, which are also important for intellectual performance. Emerging evidence suggests that dietary phytochemicals, in particular flavonoids that can be found in for example berries, may exert beneficial effects on human memory and neuro-cognitive performance [12,13].

Swedish children need to double their current intake of fruit, berries and vegetables in order to obtain sufficient fibre and vitamins according to national guidelines [14]. The consumption of fruit and vegetables in the adult population is also generally low, with only 13% of women and 5% of men meeting recommendations [15]. In this context, school fruit and vegetable schemes provide an opportunity to promote healthier options in the school food environment [16].

In a previous study, performance in children was assessed using the validated d2 test of attention, measuring attention span and concentration [17]. In order to test the hypothesis that a mid-morning beverage may affect the academic performance of schoolchildren, a smoothie rich in fruits, berries and vegetables was developed and administered to children aged 10–12 years in a school setting. The choice of design for a study such as this depends on the material, the study situation and the analyses to be made. A cross-over design is statistically efficient due to the reduction in confounding variables when each participant serves as her or his own control, while fewer subjects are also required than for a parallel study. However, parallel studies can be carried out in a shorter space of time, thereby avoiding a higher drop-out rate. Assay sensitivity is a property of a clinical trial defined as the ability to distinguish an effective treatment from a less effective or ineffective treatment [18] and is used, as in the present study, to investigate if a study design is appropriate for the detection of an assumed difference between treatments. The primary aim of the present pilot study was thus to investigate if a cross-over design was appropriate to study the effects of berry-enriched smoothie consumption on academic performance in a school setting.

## Materials and methods

### Subjects and design

In total, 250 schoolchildren in grades four and five from nine schools in southern Sweden were invited to participate in the study, of whom 236 accepted. Reasons for dropouts included language difficulties for newly arrived children/families and children changing place of residence. In total, 216 children participated in the first study period and 221 participated in the second, with seven children absent during the first period due to illness (Table 1). Cluster randomisation (units: school class groups) was performed when the children had agreed participation.

**Table 1.** Number of participants divided by gender and grade in study groups A and B for trial periods 1 and 2, respectively.

		Period 1			Period 2		
		Male	Female	Total	Male	Female	Total
Group A	Grade 4	30	38	68	30	39	69
	Grade 5	21	18	39	17	22	39
Group B	Grade 4	23	39	62	26	39	65
	Grade 5	23	24	47	24	24	48
	Total	97	119	**216**	97	124	**221**

The study was designed as a cross-over trial comprising two study periods of 10 school days each (Table 2). Children were randomly divided into two study groups, A and B, with the groups administered either an active berry smoothie (group A) or a fruit-based control beverage with the same energy content (group B). The beverages were administered just after the midmorning break at 9:30–10:00 a.m. After a three week wash-out period, group A was administered the control and group B the active smoothie. Each child was given an individual beverage carton containing 250 mL, every day. The amount of beverage consumed was determined by weighing each carton, before and after consumption. It was noted that the study engaged the children and that they discussed and compared vividly the flavours and general acceptability of the beverages.

**Table 2.** Description of the cross-over design.

	1	2	3	4	5	6	7
Week	Period 1		Washout			Period 2	
Group A	Active smoothie					Control beverage	
Average consumption (g)	173					246	
Group B	Control beverage					Active smoothie	
Average consumption (g)	248					136	
d2 test*	Test 1	Test 2				Test 3	Test 4

*The first and third tests were carried out one school day before the periods of beverage consumption; the second and fourth tests were carried out on the last day of beverage consumption.

Prior to the study, all children answered a questionnaire about their consumption of fruit, berries and vegetables, physical activity habits, breakfast consumption habits and their perceived hunger during the school day.

### Measurement of attention

An estimate of academic performance was made using Brickenkamp’s 10^th^ edition of the d2 test of attention, measuring concentration, processing speed and accuracy. The d2 test of attention is a neuropsychological measure of selective and sustained attention and visual scanning speed (19), and has proven to have good reliability and validity in assessing an individual’s ability to concentrate and pay attention, independent of intelligence [19]. The d2 test is a paper and pencil test consisting of 14 lines containing 57 lower-case letters ‘d’ and ‘p’, with a variable number of dashes (one to four) above or below the letters. Test participants are instructed to mark specified target characters (*e.g*. the letter ‘d’ with a total of 2 dashes) and to leave out surrounding distractors that are similar to targets, for example a ‘p’ with two dashes or a ‘d’ with one or three dashes. The test is individual and rapid as it is completed within 8 minutes.

In the present study the children were instructed according to the standard instructions to mark as many target characters per line as possible. Every 20 seconds, the children were instructed to move on to the next line, regardless of how far they had progressed on the previous line. Processing speed (PTO) was defined as the total number of characters processed; Concentration Performance (CP) was derived from the number of correctly crossed-out relevant items minus the errors of commission; and the total error percentage (EP) was defined as incorrectly marked distractor characters plus unmarked ‘d’s’ with two dashes, divided by processing speed.

### Active smoothie and control beverages

The active smoothie was designed from a base of pear and apple juice with the addition of various berry and vegetable purées made from local produce (for final ingredients see Table 3). In order to increase the content of healthy fatty acids and vitamin E, as well as to augment the bioavailability of *e.g*. beta carotene, oat oil was added to the active smoothie. Another requirement for product development was that smoothie texture should be such that participants were able to drink it through a straw. After evaluation of prototypes by an internal expert panel, three different smoothies were chosen for preference testing by a total of 46 schoolchildren, 27 in grade 3 and 19 in grade 5. Based on the combination of acceptance tests using a hedonic scale and ranking, the most preferred smoothie was selected and subsequently used as the active product in the study. For nutritional content and descriptive quality parameters of the smoothie and control beverage, see Table 4.

**Table 3.** List of ingredients in the active smoothie and control beverage.

Active smoothie	Content (%)	Control beverage	Content (%)
Pear juice	40.2	Water	80.0
Apple juice	13.1	Invert sugar. 73° Brix	12.4
Apple purée	10.1	Apple juice. 12.5° Brix	5.0
Strawberry purée	10.1	Pear juice concentrate. 70° Brix	2.2
Blackcurrant purée	6.5	Citric acid	0.3
Pear purée	6.5	Pear aroma	0.05
Carrot purée	6.5		
Elderberry purée	3.9		
Spinach, chopped and frozen	2.0		
Oat oil	1.0

**Table 4.** Analysed nutritional content and quality parameters of the active smoothie and the control beverage.

Nutrient and quality parameters	Active smoothie	Control beverage
Water (g/100g)	89.0	89.5
Crude protein (g/100g)	0.34	
Carbohydrates (g/100g)	10.3	10.5
Energy (kcal/kJ per 100g)	43/181	43/179
Fructose (g/100g)	5.31	3.77
Glucose (g/100g)	2.21	3.36
Sucrose (g/100g)	0.09	3.51
Total sugars (g/100g)	7.61	10.64
		
Vitamin E (alpha-tocopherol, mg/100g)	0.36	-
Vitamin C (mg/100g)	-	-
Beta-carotene (μg/100g)	421	-
		
Total phenol content (mg gallic acid/100 mL)	86.6	9.4
Total anthocyanin content (mg cyanidin-3- glucoside/100 mL)	19.1	-
		
Potassium (mg/L)	1700	340
Phosphorus (mg/L)	180	27
Calcium (mg/L)	120	110
Magnesium (mg/L)	75	25
Sulphur (mg/L)	55	20
Sodium (mg/L)	30	56
Boron (mg/L)	1.2	0.58
Iron (mg/L)	1.2	0.43
		
pH	3.7	3.0
Soluble dry matter (°Brix)	11.8	11.8
Titratable acid (g citric acid/100 mL)	0.52	0.41

The control beverage was composed of water, sugar, clear pear juice and pear aroma (Table 3), with cloudy apple juice added to make the texture of the beverage resemble that of the smoothie. Consequently, the active smoothie contained high content of bioactive components (polyphenols, carotenoids and vitamin E), and oat oil, which occurred in no or minimal amount in the control beverage. Both the active smoothie and the control beverage were standardised to the same Brix value and energy content, pasteurised (95°C, 30 s) and placed in 250 mL Tetra Brik cartons equipped with a straw.

### Ethics

School head teachers and subsequently each child’s parents gave written consent for participation in the study. The study design was given an advisory pronouncement (registration number 2014/206) by the regional ethical board, with guidelines from the Swedish Research Council [20] followed during the research process.

### Statistical analyses

Prior to the start of the trial, power calculations were performed. A total of 250 subjects were needed in order to achieve 80% power to detect a difference in PTOdiff of 2.5 units, between the effects of the active smoothie and the control beverage. The Wilcoxon signed rank test was employed to test the hypothesis that the active smoothie and control beverage were equally beneficial. Statistical analyses were performed using StatXact v 10.3.

## Results

The children consumed significantly less of the active smoothie, on average 92 grams less, compared to the control beverage (p = 0.000). Further, the overall variation in consumed amounts was lager when the children drank the active smoothie as compared to the control (154g ± 98 vs. 246 ± 28; Mean ± Std). A 63% share of the children rated the control beverage as ‘very tasty’ on a hedonic scale as compared to only 18% for the active smoothie.

Table 5 shows differences in processing speed, *i.e*. the processed number of target objects (PTO), between group A (active) and group B (control). Both treatments resulted in a statistically significant increase in PTO during the test period (p = 0.000), with no statistically detectable difference in performance change between treatment groups (p = 0.459).

**Table 5.** Change in processing speed (processed number of targets, PTO) following consumption of the active smoothie and the control beverage.

	Active smoothie			Control beverage			
Statistica	Start	End	Change	Start	End	Change	Change Active – Control
N	226	220	210	232	217	214	192
Missing	10	16	26	4	19	22	44
Mean	147.5	165.7	19.4	144.8	167.1	22.8	−2.98
Std	40.3	44.0	18.8	33.9	40.2	24.1	27.1
P-value^1^	NA	NA	0.000	NA	NA	0.000	0.459
95% CI LL	142.17	159.87	16.89	140.45	161.74	19.55	−6.83
95% CI UL	152.75	171.55	21.99	149.21	172.49	26.04	0.87

a Wilcoxon signed rank test. 2-sided p-value.

Table 6 shows the differences in concentration performance (CP) between group A (active) and group B (control). Both treatments resulted in a statistically significant increase in CP during the test period (p = 0.000), with again no statistically detectable difference recorded in performance change between treatment groups (p = 0.938).

**Table 6.** Change in concentration performance (CP) following consumption of the active smoothie and the control beverage.

	Active smoothie			Control beverage			
Statistica	Start	End	Change	Start	End	Change	Change Active – Control
N	226	220	210	232	217	214	192
Missing	10	16	26	4	19	22	44
Mean	129.4	147.6	19.2	127.4	147.4	20.0	−1.0
Std	39.8	42.9	17.5	33.1	37.3	18.7	26.0
P-value^a^	NA	NA	0.000	NA	NA	0.000	0.938
95% CI LL	39.90	34.64	−8.67	38.93	37.51	−3.97	−11.87
95% CI UL	54.54	48.80	−1.11	52.68	53.05	5.97	1.65

a Wilcoxon signed rank test. 2-sided p-value.

Regarding the error percentage (EP) results shown in Table 7, a significant change was noted during the active period (reduction, p = 0.014) but not during the control period (p = 0.202). However, the results from both the active group and the control group were not significant.

**Table 7.** Change in error percentage (EP) following consumption of the active smoothie and the control beverage.

	Active smoothie			Control beverage			
Statistica	Start	End	Change	Start	End	Change	Change Active – Control
N	226	220	210	232	217	214	192
Missing	10	16	26	4	19	22	44
Mean	47.2	41.7	−4.9	45.8	45.3	1.0	−5.1
Std	55.8	53.3	27.8	53.2	58.1	36.9	47.5
P-value^3^	NA	NA	0.014	NA	NA	0.202	0.915
95% CI LL	39.90	34.64	−8.67	38.93	37.51	−3.97	−11.87
95% CI UL	54.54	48.80	−1.11	52.68	53.05	5.97	1.65

a Wilcoxon signed rank test. 2-sided p-value.

## Discussion

The aim of the present work was to investigate if a conventional cross-over design could be used to study the effects of smoothie consumption on academic performance in a school setting, *i.e*. if the design possessed assay sensitivity [18]. Three components of typical smoothies, water, energy and the bioactive compounds derived from berries, fruits and vegetables, could hypothetically play a role in improving children’s academic performance [1,4,11,12,21]. In our study, the results obtained from the d2 test of attention indicated an effect of water and energy supplementation, with both groups exhibiting an increase in performance – measured as speed (PTO) and concentration (CP) – following the consumption of both the active and control beverages. A tendency towards a decreased number of errors (EP) was noted after consumption of the active smoothie in comparison to the control beverage. Interestingly, this effect was evident even though the children consumed approximately 40% less of the active smoothie compared to the control. Thus, the d2 test is adequately sensitive to detect differences due to exposure. In this regard, the cross-over design also seems to be valid.

However, the d2 test in combination with the cross-over design did not produce any conclusive results regarding the effect of the bioactive compounds consumed on academic performance. Two major problems with the research design were identified – differences in sensory quality of the products and decreased power compared to the plan.

It was challenging for many of the children to accept the somewhat unfamiliar texture and flavour of the active smoothie, characterised by dark berries such as blackcurrant and elderberry. This may in itself reflect an overall low berry consumption among Swedish children [14], who are not used to more astringent beverages. A recent Danish study has further shown that children prefer yellowish-brown juices over those of a red colour [22]. Another study, looking at exposure effects when children aged 9–11 years are repeatedly exposed to fruit juices, found that these effects were influenced by initial liking; although ‘initial dislikers’ benefitted most from repeated exposure, their liking and intake did not reach that of ‘initial likers’ across eight exposures [23]. When designing future studies, even more attention should be paid to sensory characteristics in order to avoid these dilemmas. In a coming full size study it is important to design the smoothies so they are more similar in texture and taste.

Another weakness in this pilot study is the decreased power compared to the power in the design phase of the trial. A higher power can be achieved by larger number of classes, or by randomisation at an individual level instead of cluster randomisation. A more similar level of consumption might also help to detect possible differences in outcome. Thus, this pilot study has shown that although the d2 test and study design were adequate in measuring the effect of a beverage containing water and energy on performance, the employed set up did not allow for the detection of a more subtle effect of bioactive compounds.

Future research could instead be designed, using a larger sample size, as a parallel study, providing more robust results and requiring only one study period. The effect of the bioactive compounds might further be more readily detected after an extended study period, with a third group, receiving no beverage, included in an improved study protocol aimed at identifying the cause of this effect.

The results of the present pilot study illustrate the methodological challenges encountered when performing this type of research in a school setting. Despite accommodating teachers and head teachers, the design of the study, for logistical reasons, had to be rigorously adapted due to organisational and curricular considerations. Further, it was clear that the children influenced each other with regard to liking and consuming the beverages. This is in accordance with a recent study by Andersen et al., which found that although classmates influenced each other’s ranking of a new type of school meal, they did not influence rankings of familiar lunch packs [24]. The results stress the social dimension of liking and show that social mechanisms are very important when implementing new health-promoting food initiatives in schools [24]. Finally, another new Danish study, resembling ours in that it used the d2 test of attention but focusing on the effects of whole-meal breakfasts provided to school children, points at similar challenges as ours in interventions that are conducted in the open and dynamic school environment [25]. Thus, the result of this pilot may be useful in designing other studies with ecological validity.

## Conclusions

Pupil academic performance in the d2 test of attention was positively affected by beverage consumption, a finding attributed to the supplementation of water and energy. Application of the proposed study design produced no conclusive results regarding the effect of bioactive compounds on concentration; although a significant decrease in error percentage was found in the active group but not in the control group, the difference between the groups was not significant. Thus, it could not be proven that the employed cross-over design possesses assay sensitivity. Future work should include a third group, receiving no beverage, in order to identify the cause of the effect, while a parallel-study design should also be considered. Further, the results of this pilot clearly stress both the challenges coupled to, and significance of, sensory factors in designing trials aimed at investigating effects of beverages and foods in children.
